# Stable retention of chloramphenicol-resistant mtDNA to rescue metabolically impaired cells

**DOI:** 10.1038/s41598-020-71199-0

**Published:** 2020-08-31

**Authors:** Emma R. Dawson, Alexander N. Patananan, Alexander J. Sercel, Michael A. Teitell

**Affiliations:** 1 Department of Pathology and Laboratory Medicine, University of California, Los Angeles, CA 90095 USA; 2 Molecular Biology Interdepartmental Program, University of California, Los Angeles, Los Angeles, CA 90095 USA; 3 Eli and Edythe Broad Center of Regenerative Medicine and Stem Cell Research, University of California, Los Angeles, Los Angeles, CA 90095 USA; 4 California NanoSystems Institute, University of California, Los Angeles, Los Angeles, CA 90095 USA; 5 Department of Pediatrics, David Geffen School of Medicine, University of California, Los Angeles, Los Angeles, CA 90095 USA; 6 Jonsson Comprehensive Cancer Center, David Geffen School of Medicine, University of California, Los Angeles, Los Angeles, CA 90095 USA

**Keywords:** Mitochondria, Energy metabolism

## Abstract

The permanent transfer of specific mtDNA sequences into mammalian cells could generate improved models of mtDNA disease and support future cell-based therapies. Previous studies documented multiple biochemical changes in recipient cells shortly after mtDNA transfer, but the long-term retention and function of transferred mtDNA remains unknown. Here, we evaluate mtDNA retention in new host cells using ‘MitoPunch’, a device that transfers isolated mitochondria into mouse and human cells. We show that newly introduced mtDNA is stably retained in mtDNA-deficient (ρ0) recipient cells following uridine-free selection, although exogenous mtDNA is lost from metabolically impaired, mtDNA-intact (ρ+) cells. We then introduced a second selective pressure by transferring chloramphenicol-resistant mitochondria into chloramphenicol-sensitive, metabolically impaired ρ+ mouse cybrid cells. Following double selection, recipient cells with mismatched nuclear (nDNA) and mitochondrial (mtDNA) genomes retained transferred mtDNA, which replaced the endogenous mutant mtDNA and improved cell respiration. However, recipient cells with matched mtDNA-nDNA failed to retain transferred mtDNA and sustained impaired respiration. Our results suggest that exogenous mtDNA retention in metabolically impaired ρ+ recipients depends on the degree of recipient mtDNA-nDNA co-evolution. Uncovering factors that stabilize exogenous mtDNA integration will improve our understanding of in vivo mitochondrial transfer and the interplay between mitochondrial and nuclear genomes.

## Introduction

Mutations in the multi-copy mitochondrial genome (mtDNA) can impair the biosynthesis of ATP, metabolites, fatty acids, reactive oxygen species, and iron sulfur clusters^1–4^. Even a single nucleotide polymorphism can have profound effects on cellular function and contribute to pathologies including cardiomyopathies, diabetes, autoimmune diseases, neurological disorders, cancer, and even aging^5,6^. The degree of pathology often depends on the ratio of mutant to wild-type mtDNA populations within the same cell, a situation known as heteroplasmy^7^. One in 5,000 people have some degree of a pathological mtDNA disorder, and up to 1 in 8 individuals carry low levels of a mtDNA mutation that can be inherited through the maternal germline^8–11^. Mitochondrial replacement therapy (MRT) aims to prevent transmission of mtDNA disorders from affected mothers to offspring, but limited treatments exist for those already living with a pathological mtDNA mutation^12,13^.

Our ability to repair mutant mtDNA and improve metabolically impaired cells would advance disease modeling studies and potential cell-based therapies for mtDNA disorders. Gene therapy and now gene editing is a viable treatment option for some nucleus-encoded disorders^5,14,15^. In contrast, specific mtDNA mutations are difficult to generate or repair because current gene modifying approaches do not work well inside mitochondria. Zinc finger nucleases (ZFNs) and transcription activator-like effector nucleases (TALENs) target and degrade detrimental mtDNAs both in vitro and in vivo, shifting heteroplasmy ratios. However, these modifiers can be challenging to engineer, only degrade pre-existing target mtDNAs, are inefficient with incomplete removal of target mtDNAs, and they cannot generate new mtDNA sequences inside cells^16–23^. To bypass most of these issues, the transfer of mitochondria containing desired mtDNA sequences into cells of interest can generate desirable hybrid cells with unique mtDNA-nDNA pairings. Current mitochondrial transfer approaches for somatic cells include MitoCeption^24^, microinjection^25^, cell fusion^26^, co-culturing^27,28^, isolated mitochondrial co-incubation^29^, magnetomitotransfer^30^, and large cargo delivery platforms^31^. These techniques have in common the provision of mitochondria containing exogenous mtDNA into mtDNA-deficient (ρ0) recipient cells, often followed by selection in uridine-deficient culture medium^32^. ρ0 cells are typically generated using DNA intercalating drugs, such as ethidium bromide, or DNA polymerase chain terminators, such as 2′,3′- dideoxycytidine, to remove recipient cell mtDNA^33,34^. However, these drugs can cause off target nDNA mutations and are not equally effective in removing all endogenous mtDNA from all cell types. In addition, ρ0 mammalian cells do not naturally exist, leading to questions about physiological relevance. An ability to transfer isolated mitochondria and retain exogenous mtDNA in unmodified, endogenous mtDNA containing (ρ+) recipient cells would alleviate many of these potential concerns.

We recently developed a simple mechanical force based hardware device called ‘MitoPunch’ to transfer isolated mitochondria into mammalian cells. Here, we used MitoPunch to transfer chloramphenicol-resistant (CAP-R) mitochondria into chloramphenicol-sensitive ρ+ recipient cybrid cells that contain mutant mtDNA with impaired respiration. We evaluated whether introduced CAP-R mtDNA into ρ+ recipient cybrid cells was retained or transient and lost when the recipient cell nDNA matched and co-evolved, or was mismatched, with the cybrid cell mtDNA strain, and the resultant effect on respiratory function.

## Results

### MitoPunch transfer of mitochondria into ρ0 cells

**Figure 1:**
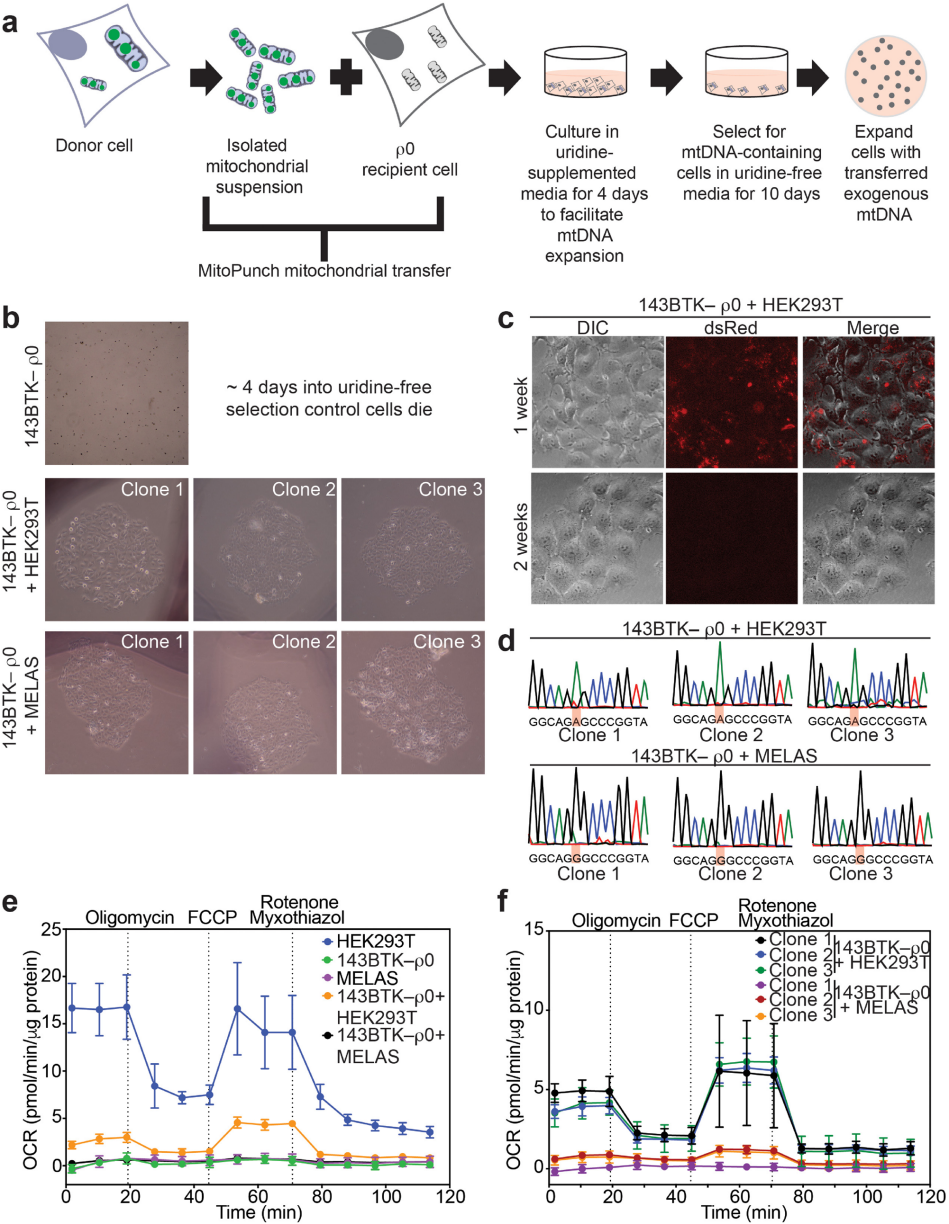
Stable mitochondrial integration in ρ0 cells. (**a**) Schematic showing selection of ρ0 cell with successfully retained exogenous mtDNA. (**b**) 143BTK- ρ0 cells with transferred HEK293T or MELAS A3243G mitochondria were selected on uridine-deficient media. Approximately 2 weeks after mitochondrial transfer, colonies were imaged on an inverted microscope and 5 × objective. (**c**) 143BTK- ρ0 + dsRed- labeled HEK293T mitochondria were visualized by DIC and fluorescence microscopy 1 and 2 weeks after mitochondrial transfer. (**d**) Sanger sequencing of 3 clones derived from 143BTK− ρ0 cells transferred HEK293T or MELAS mitochondria. Orange highlight denotes mtDNA position 3243. (**e**) Seahorse Extracellular Flux analysis to quantify oxygen consumption rate of bulk culture generated from 143BTK− ρ0 cells transferred HEK293T or MELAS mitochondria. (**f**) Seahorse Extracellular Flux analysis to quantify oxygen consumption rate of clones generated from **(e)**. (**e, f**) Oligomycin, FCCP, and rotenone/myxothiazol are an ATP synthase inhibitor, uncoupler, and complex I/III inhibitors, respectively. Each data point represents the average of 3 technical replicates and the error bar denotes standard deviation.

To begin, we used MitoPunch to transfer isolated mitochondria into ρ0 cells, to evaluate the reacquisition of respiratory function or to generate a model of mtDNA disease in a cell system that prior studies indicated should work^24–28,31,35^. ρ0 cells lack a functional electron transport chain (ETC), which blocks dihydroorotate dehydrogenase enzymatic activity and stops endogenous pyrimidine biosynthesis, leading to cell death with time^33,36,37^. Thus, ρ0 cells can only persist in vitro in uridine-supplemented media or in uridine-deficient medium when they reacquire ETC activity. Isolated dsRed-labeled HEK293T^38^ or mitochondrial encephalopathy, lactic acidosis, and stroke-like episodes (MELAS) A3243G cybrid^39^ mitochondria were MitoPunch transferred into 143BTK−  ρ0 osteosarcoma cells in an attempt to generate 143BTK−  ρ0 + HEK293T or 143BTK−  ρ0 + MELAS hybrid cells, respectively (Fig. 1a). Post-transfer, we grew cells for 4 days in uridine-replete media for recovery, followed by a shift to 10 days of uridine-deficient growth conditions to select for cells with reacquired ETC activity (Fig. 1b). The presence of undisrupted donor cells was minimized by introducing additional centrifugation spins in the mitochondrial isolation procedure and by passing the mitochondrial isolate through a 3 μm filter before reaching the recipient cells, which is an indirect benefit of the MitoPunch transfer pipeline. We isolated three clones from 143BTK− ρ0 + HEK293T or 143BTK− ρ0 + MELAS bulk cultures that contained hundreds or tens of independent colonies, respectively. Since 143BTK−  ρ0 + HEK293T cells received dsRed-labeled mitochondria, we could observe the turnover of transferred mitochondria over time, with the label disappearing between one and two weeks after mitochondrial transfer (Fig. 1c). This observation further confirms that there was no whole cell contamination from the mitochondrial donor and is consistent with the predicted turnover rate for mitochondrial proteins^40,41^. Following the 10 day uridine-deficient selection, clones were isolated and expanded. Two months post-transfer, when no original HEK293T or MELAS mitochondrial proteins remained, sequencing of three independent clones of each new hybrid cell type showed persistence of the exogenous mtDNA (Fig. 1d). To assess mitochondrial function, we measured the oxygen consumption rate (OCR) for each bulk culture and each of the six individual clones (Fig. 1e,f). HEK293T cells have a robust respiratory profile, while the 143BTK− ρ0 and MELAS cells have abolished respiration. Compared to 143BTK−  ρ0 cells with abolished respiration, 143BTK−  ρ0 + HEK293T hybrid cells showed an improved respiratory profile. In contrast, 143BTK−  ρ0 + MELAS hybrid cells recapitulated the impaired respiratory profile observed for MELAS patient-derived cells^12,42^. Additionally, we performed qPCR on 143BTK− ρ0 cells containing either MELAS or wild type (WT) transferred mitochondria to compare the restored mtDNA levels to unmodified 143BTK− parental cells (Supplementary Fig. S1). After several weeks of cell culture and freeze–thaw cycles, 143BTK− ρ0 + WT transfers showed mtDNA copy numbers comparable to 143BTK− parent cells. However, 143BTK− ρ0 + MELAS cells maintain a slightly lower mtDNA copy number. In sum, the MitoPunch mitochondrial transfer and selection pipeline yields permanently retained exogenous mtDNA in a ρ0 recipient cell type, as anticipated, and can model defective respiration that characterizes a typically severe mtDNA disease.

### Transferred WT mtDNA is lost from ρ + MELAS cells

**Figure 2:**
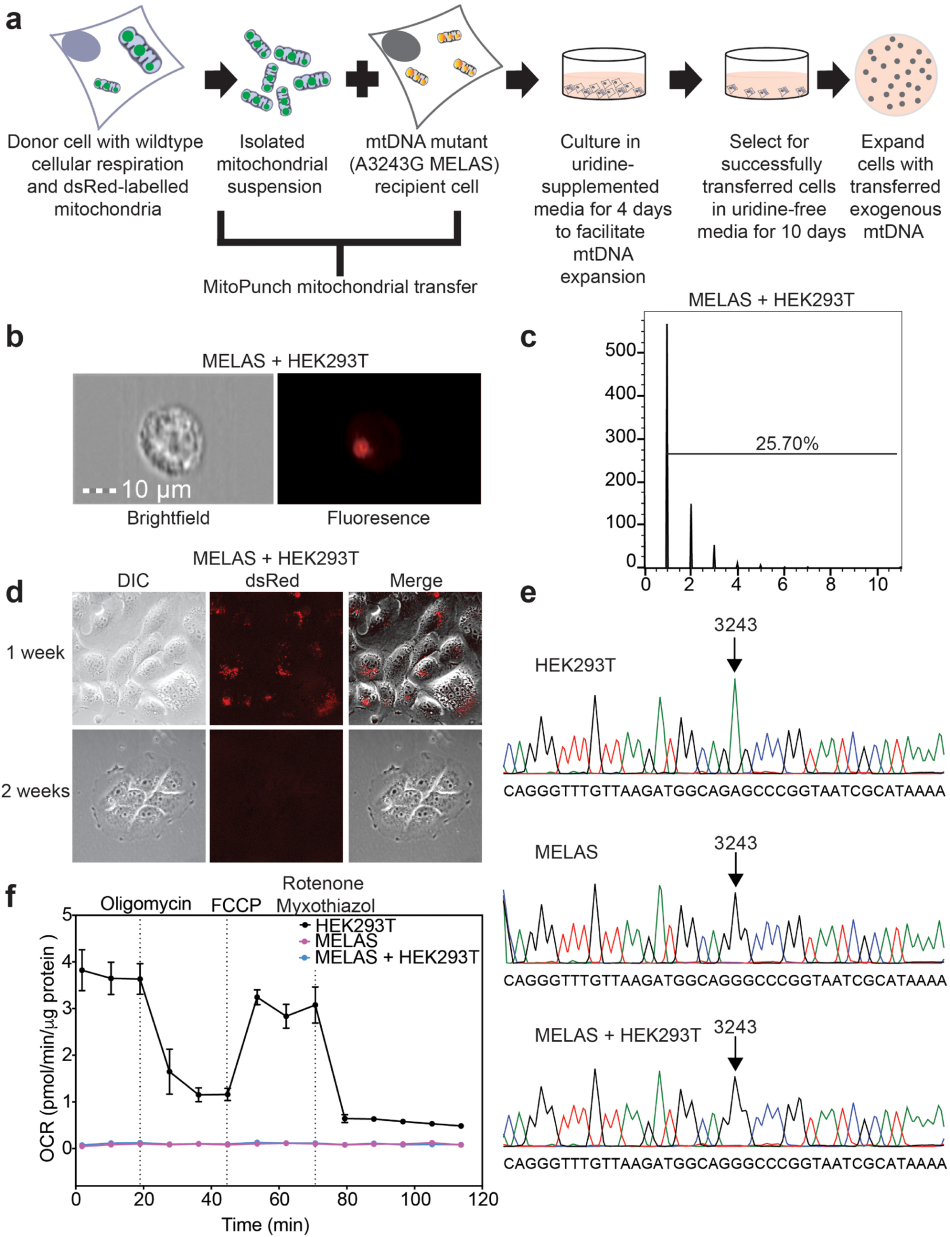
Transfer of functional mtDNA is not maintained in ρ + mutant cells. (**a**) Schematic showing selection of ρ + mutant cell with transferred exogenous mtDNA. (**b,c**) Isolated dsRed-labeled HEK293T mitochondria were transferred by MitoPunch into MELAS cybrid cells and immediately analyzed by ImageStream. Brightfield and fluorescence data was collected for 10,000 cells. The number of transferred mitochondria was quantified for each cell. (**d**) MELAS + HEK293T were visualized by DIC and fluorescence microscopy 1 and 2 weeks after mitochondrial transfer. (**e**) Sanger sequencing of HEK293T, MELAS, and MELAS + HEK293T cells. Arrows denote mtDNA position 3243. (**f**) Seahorse Extracellular Flux analysis to quantify oxygen consumption rate of HEK293T, MELAS, and MELAS + HEK293T cells. Oligomycin, FCCP, and rotenone/myxothiazol are an ATP synthase inhibitor, uncoupler, and complex I/III inhibitor, respectively. Each data point represents the average of 3 technical replicates and the error bar denotes standard deviation.

We next examined whether MitoPunch transfer could improve the mitochondrial function of ρ + (endogenous mtDNA containing) recipient cells with impaired respiration. As above, we performed MitoPunch transfer of isolated dsRed-labeled HEK293T mitochondria this time into human cybrid cells containing an A3243G MELAS mtDNA mutation (Fig. 2a). Immediately following MitoPunch, we visualized potential MELAS + HEK293T hybrid cells using ImageStream flow cytometry (Fig. 2b) to assess the number of recipient cells with exogenous mitochondria and the number of dsRed-labeled mitochondrial “speckles” per cell. ImageStream data showed that ~ 25% of MitoPunch recipient MELAS cybrid cells acquired 1–6 dsRed speckles per cell, providing a crude estimate of mitochondria transferred (Fig. 2c). We performed an independent experiment and again applied uridine-deficient media selection because MELAS cybrid cells show markedly impaired cellular respiration (Fig. 1e)^24,26,31,35^. Similar to ρ0 recipient cells, exogenous HEK293T mitochondrial proteins remain for one to two weeks post-transfer in selection media (Fig. 2d). However, unlike ρ0 recipients, MELAS cells do not retain exogenous mtDNA beyond 2 months post-transfer, as shown by the continued presence of only the A3243G mtDNA sequence for MELAS + HEK293T bulk cultures containing tens of colonies (Fig. 2e).

We examined a second, independent, MELAS cybrid cell recipient (MELAS2), homoplasmic for A3243G mtDNA, by transferring WT functional mitochondria isolated from donor cells obtained from the same individual. MitoPunch transfer cells underwent selection for four weeks in uridine-deficient media with ~ 50 independent MELAS2 + WT colonies obtained (Supplementary Fig. S1). However, a similar number of independent colonies were also obtained for MitoPunch transfer cells that received 1 × phosphate buffer saline (PBS), pH 7.4, indicating that very low level ETC function in MELAS cybrids is sufficient for survival in uridine-free media selection. We anticipate that ~ 25% of recipient cells obtained WT mtDNA similar to MELAS + HEK293T cells (Fig. 2b,c). However, MELAS2 + WT cells also did not show evidence for exogenous mtDNA after four weeks of selection by sequencing the bulk culture containing ~ 50 independent colonies (Supplementary Fig. S1).

We measured the OCR of MELAS + HEK293T cells in bulk cultures containing tens of independent colonies as a second assessment of retained exogenous mtDNA one month post-transfer. In contrast to transfers into 143BTK−  ρ0 recipient cells, MELAS + HEK293T cells replicated the impaired respiratory profile characteristic of the parent MELAS cybrid cells without improved respiration (Fig. 2f). In addition, when isolated MELAS and HEK293T mitochondria were mixed at 1:1 or 10:1 ratios and then MitoPunch transferred into 143BTK−  ρ0 recipient cells, we observed significant MELAS mtDNA retention in the 10:1 mixture in addition to the anticipated retention of HEK293T mtDNA (Supplementary Fig. S1). This indicates that the MitoPunch transfer and selection pipeline can generate heteroplasmic clones in addition to homoplasmic clones that may resemble certain physiologic conditions, at least in certain ρ0 recipient cells. Increasing the MELAS mtDNA population relative to WT mtDNA also resulted in increasingly impaired respiration, as anticipated for an increasingly mutant mtDNA heteroplasmic state (Supplementary Fig. S1). Overall, these data indicate that two independent MELAS recipient cells examined here do not retain exogenous mtDNA that can potentially improve respiration. This is different from 143BTK−  ρ0 cells (Fig. 1, Supplementary Fig. S1) and suggests strong selective pressure to remove exogenous mtDNA and retain endogenous mutant mtDNA in these ρ + recipients despite potential respiratory advantages for retaining WT mtDNA.

### Transfer of CAP-R mtDNA confers resistance to Δmt-ND4 cells

**Figure 3:**
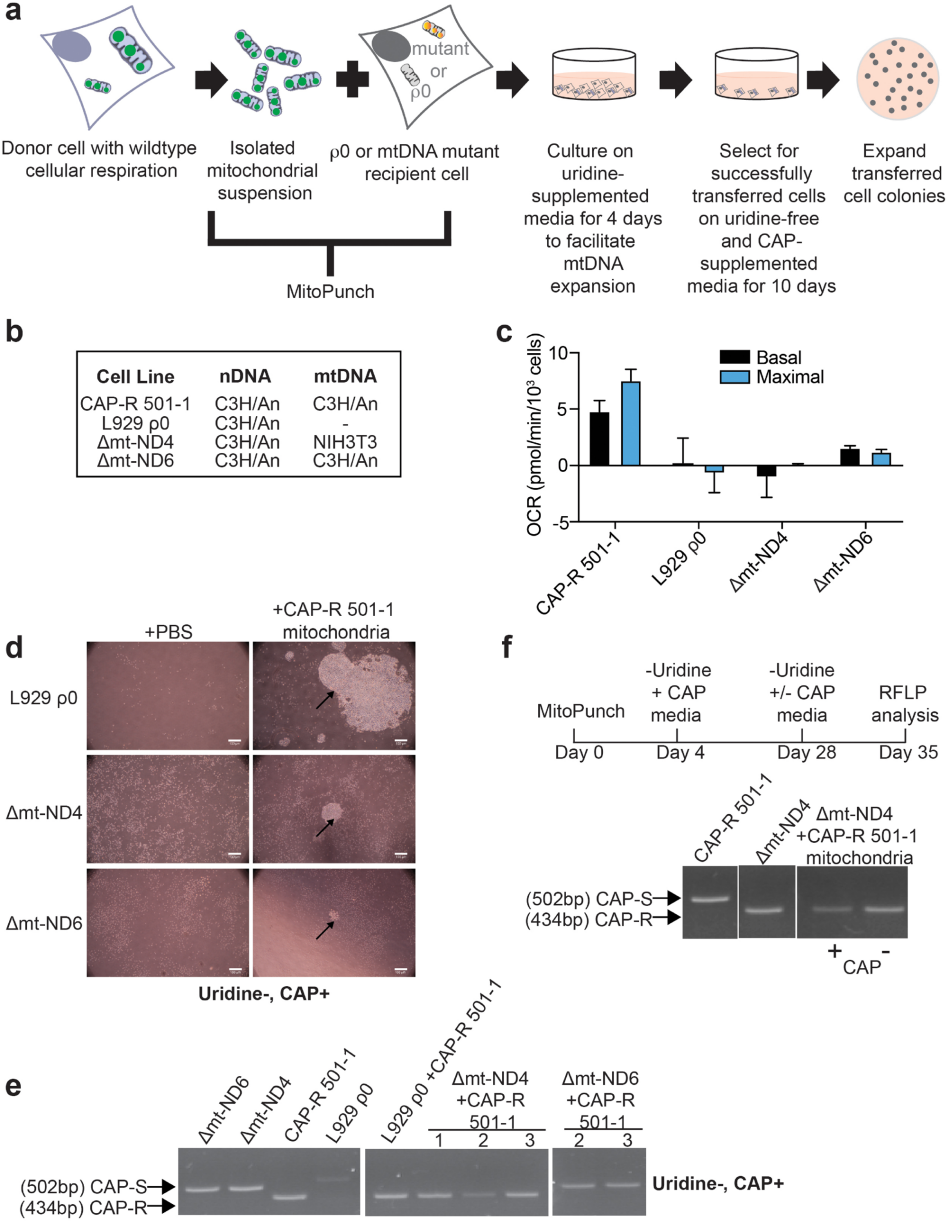
Chloramphenicol selection for transferred CAP-R mtDNA retention. (**a**) Selection of mouse ρ0 or ρ + mutant cells with successfully retained exogenous CAP-R 501-1 mtDNA. (**b**) Cell lines used with known nuclear and mitochondrial mouse backgrounds. (**c**) Seahorse Extracellular Flux analysis quantification of basal and maximal cellular respiration in Δmt-ND4, Δmt-ND6, CAP-R 501-1, and L929 ρ0 cells. Two-tailed, unpaired Student’s t-test comparing samples to L929ρ0. * represents significance with * < 0.05, ** < 0.01, *** < 0.001, **** < 0.0001. Black * represents significance for Basal Respiration and Blue * represents significance for Maximal Respiration. The bar height denotes average of 3 replicates and the error bars are the standard deviation. (**d**) Phosphate buffered saline (PBS) or CAP-R 501-1 mitochondria were transferred into L929 ρ0, Δmt-ND4, and Δmt-ND6 recipient cells and were selected on uridine-deficient, CAP-supplemented media. Four weeks after mitochondrial transfer, colonies were imaged with an inverted microscope and 5 × objective. Scale bar denotes 100 µm. (**e**) RFLP analysis of CAP-R 501-1, L929 ρ0, L929 ρ0 + CAP-R 501-1, Δmt-ND4 + CAP-R 501-1, and Δmt-ND6 + CAP-R 501-1 bulk culture cells two weeks after mitochondrial transfer. (**f**) Following Δmt-ND4 + CAP-R 501-1 mitochondrial transfer, cells were cultured in (1) uridine-supplemented media for four days, (2) uridine-deficient, CAP-supplemented media for 24 days, and (3) uridine-supplemented media with or without CAP for 7 days. RFLP analysis of CAP-R 501-1, Δmt-ND4, and Δmt-ND4 + CAP-R 501-1 mitochondria. In **(e–f)**, arrows denote the difference between CAP-S (502 bp) and CAP-R (434 bp) PCR products post-MaeII digestion on a 2.5% agarose gel electrophoresis. CAP-R 501-1 control is the same in each panel. Each of these panels were cropped from different parts of the same gel with the same exposure level.

We addressed the inability of our standard transfer and selection protocol to isolate ρ + cells with stable exogenous mtDNA by using an antibiotic-resistant mitochondrial donor to apply additional selective pressure for exogenous mtDNA retention. Prior studies generated and used mtDNA mutations that confer resistance to the mitochondrial translation inhibitor, chloramphenicol (CAP). CAP-resistant (CAP-R) mitochondria first showed utility for mitochondrial transfer by microinjection and cybridization with CAP-S cells having WT respiratory profiles^25,43–45^. Since these studies showed exogenous mtDNA retention in WT ρ + cells, we examined whether this would work with our MitoPunch pipeline to permanently improve mitochondrial function in ρ + mutant cells (Fig. 3a). We used mouse fibroblast cell line CAP-R 501-1, which contains a mtDNA T2433C substitution resulting in chloramphenicol-resistance, as a mitochondrial donor. CAP-R 501-1 was derived from L929 mice with co-evolved C3H/An nucleus and mitochondrial haplotypes (Fig. 3b). In addition to antibiotic resistance, CAP-R 501-1 cells show increased OCR compared to the abolished OCR in L929 ρ0 fibroblasts, but less basal respiration, maximal respiration, and mitochondrial-derived ATP production compared to the unmodified L929 parental cells (Fig. 3c, Supplementary Fig. S3). To establish that this mitochondrial donor will work in a ρ0 background, we MitoPunch transferred isolated CAP-R 501-1 mitochondria into mouse L929 ρ0 cells (L929 ρ0 + CAP-R 501-1) that were grown in uridine-deficient, CAP-supplemented media (Fig. 3a). Four weeks post-transfer and sequential selection, tens of colonies were observed (Fig. 3d) and assessed as a bulk culture by restriction fragment length polymorphism (RFLP) analyses for retention of the CAP-R 501-1 mtDNA (Fig. 3e). L929 ρ0 + CAP-R 501-1 stably integrated the exogenous CAP-R mtDNA as shown by a 434 bp PCR product that results from cleavage by MaeII only when there is a T2433C substitution^46^.

To attempt to extend this result for ρ+ mutant cells, we used two independent ρ+ recipient cybrid cells generated with different nuclear and mitochondrial DNA origins. A recent study showed that nucleus-mitochondrial genome (mtDNA-nDNA) interactions control mtDNA heteroplasmy^47^. Therefore, we tested whether recipient cells with mismatched or matched endogenous mtDNA-nDNA pair origins integrate exogenous mtDNA. For this, we used a recipient mouse cybrid cell line with a defect in complex 1 NADH dehydrogenase subunit 4 (delA10227, Δmt-ND4). Δmt-ND4 is a mismatched recipient cell line that originated from a cytoplasmic fusion between an L929 ρ0 cell (C3H/An mouse strain) and an enucleated cytoplast from the NIH3T3 mouse strain containing this deletion mutation^48^. We also used a second independent recipient mouse cybrid cell line with a defect in complex 1 NADH dehydrogenase subunit 6 (iC13887, Δmt-ND6). Δmt-ND6 is a matched recipient cell line that originated from a cytoplasmic fusion between an L929 ρ0 cell (C3H/An mouse strain) and an enucleated cytoplast from the L929 parental cell line (C3H/An mouse strain) containing this insertion mutation^49,50^ (Fig. 3b). Both Δmt-ND4 and Δmt-ND6 recipient cells have severely impaired basal and maximal respiration in contrast to a robust respiratory profile for CAP-R 501–1 mitochondrial donor cells (Fig. 3c).

We MitoPunch transferred isolated CAP-R 501-1 mitochondria into Δmt-ND4 (Δmt-ND4 + CAP-R 501-1) and Δmt-ND6 (Δmt-ND6 + CAP-R 501-1) recipient cells. Following two weeks of sequential selection in uridine-deficient, CAP-supplemented media, up to 10 colonies were obtained (Fig. 3d). RFLP analysis of the bulk culture showed that Δmt-ND4 + CAP-R 501-1 cells retained exogenous CAP-R 501-1 mtDNA four weeks after mitochondrial transfer with an undetectable level of endogenous mtDNA (Fig. 3e, Supplementary Fig. S2). We were surprised by this result because Δmt-ND4 and CAP-R 501-1 are not of the same mitochondrial origins (Fig. 3b). Our data show that endogenous mutant mtDNA was completely replaced by a mtDNA sequence of interest using an additional selection step and without making cells ρ0 first. To address stability, following four weeks on uridine-deficient, CAP-supplemented media, Δmt-ND4 + CAP-R 501-1 cells were grown with or without CAP for one additional week (Fig. 3f, Supplementary Fig. S2). RFLP analyses of the bulk culture again showed no endogenous CAP-S mtDNA and instead exogenous CAP-R mtDNA five weeks after mitochondrial transfer. Thus, exogenous mtDNA stabilized in Δmt-ND4 cells without ongoing antibiotic selection, indicating permanent mtDNA replacement. In contrast, however, Δmt-ND6 + CAP-R 501-1 bulk cultures did not retain exogenous mtDNA by RFLP analyses (Fig. 3e, Supplementary Fig. S2), even though nucleus and mitochondrial origins were the same, another unanticipated result (Fig. 3b).

### Stable transfer of CAP-R mtDNA restores respiration in ρ0 and Δmt-ND4 cells

**Figure 4:**
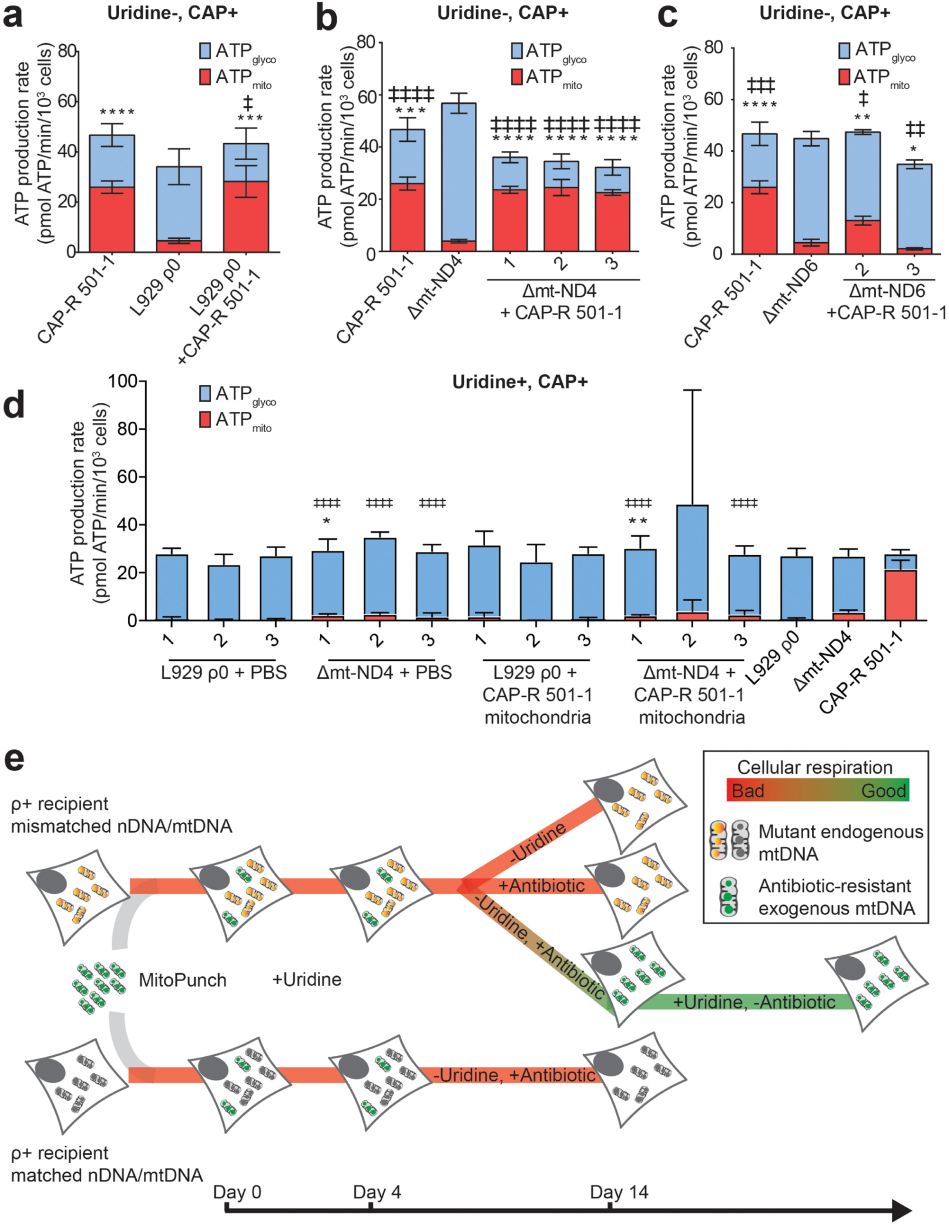
ρ0 and ρ + mutant recipient cells have restored respiration with transferred CAP-R mitochondria. (**a–c**) Seahorse Extracellular Flux analysis and quantification of ATP levels contributed by mitochondria (ATPmito) and glycolysis (ATPglyco). Cells were cultured in uridine-deficient, CAP-supplemented media. (**a**) Analysis of CAP-R 501-1 mitochondrial donor, L929 ρ0 recipient, and L929 ρ0 + CAP-R 501-1 cells. (**b**) Analysis of CAP-R 501-1 mitochondrial donor, Δmt-ND4, and Δmt-ND4 + CAP-R 501-1 bulk culture cells from three independent transfers. (**c**) Analysis of Δmt-ND6, CAP-R 501-1, and Δmt-ND6 + CAP-R 501-1 bulk cultures from two independent transfers. (**a–c**) Two-tailed, unpaired Student’s t-test comparing samples to L929 ρ0, Δmt-ND4, or Δmt-ND6. * represents significance for ATPmito and ‡ represents significance for ATPglyco. * < 0.05, ** < 0.01, *** < 0.001, **** < 0.0001. ‡ < 0.05, ‡ ‡ < 0.01, ‡ ‡ ‡ < 0.001, ‡ ‡ ‡ ‡ < 0.0001. The bar height denotes average of 4 replicates and the error bars are the standard deviation. (**d**) Seahorse Extracellular Flux analysis and quantification of ATPmito and ATPglyco in L929 ρ0, Δmt-ND4, CAP-R 501-1, L929 ρ0 + CAP-R 501-1 bulk cultures from three independent transfers, and Δmt-ND4 + CAP-R 501-1 bulk cultures from three independent transfers. Cells were cultured in uridine-supplemented, CAP-supplemented media. Two-tailed, unpaired Student’s t-test comparing samples to Δmt-ND4. * represents significance for ATPmito and ‡ represents significance for ATPglyco. * < 0.05, ** < 0.01, *** < 0.001, **** < 0.0001. ‡ < 0.05, ‡ ‡ < 0.01, ‡ ‡ ‡ < 0.001, ‡ ‡ ‡ ‡ < 0.0001. There were no statistically significant differences when comparing the samples to L929 ρ0 cells. The bar height denotes average of 5 replicates and the error bars are the standard deviation. (**e**) Schematic showing summary of ρ + mitochondrial transfer efficiency in a given selection condition.

Following permanent retention of exogenous CAP-R 501-1 mtDNA in L929 ρ0 and Δmt-ND4 ρ+ cells (Fig. 3e), we assessed changes in mitochondrial function. For this, we measured mitochondrial (ATPmito) and glycolytic (ATPglyco) ATP production using the Seahorse Extracellular Flux Analyzer. L929 ρ0 + CAP-R 501-1 bulk culture cells recovered ATPmito, basal and maximal respiration, in contrast to L929 ρ0 cells at levels comparable to CAP-R 501-1 parent donor cells (Fig. 4a, Supplementary Fig. S3). Repression of ATPglyco also accompanied increased ATPmito, basal and maximal respiration in Δmt-ND4 + CAP-R 501-1 bulk culture cells (Fig. 4b, Supplementary Fig. S3). In addition, the Δmt-ND4 + CAP-R 501-1 respiratory profile was comparable to the CAP-R 501-1 parent mitochondrial donor. However, Δmt-ND6 + CAP-R 501-1 bulk culture cells did not restore ATPmito, basal or maximal respiration (Fig. 4c, Supplementary Fig. S3). A lack of improvement in mitochondrial function in Δmt-ND6 + CAP-R 501-1 cells is likely from failed replacement of mutant mtDNA without exogenous mtDNA integration.

We note that in our studies Δmt-ND4 + CAP-R 501-1 mitochondrial transfer is only efficient with serial selection in uridine-deficient and CAP-supplemented media. Without double selection, or only CAP-supplemented media, exogenous CAP-R 501-1 mtDNA does not stably integrate into either the respiratory-incompetent L929 ρ0 or Δmt-ND4 ρ+ recipient cells as observed by continued lack of oxygen consumption (Fig. 4d, Supplementary Fig. S3). Currently, to transfer and permanently retain exogenous mtDNA in a ρ+ , mutant recipient cell, our pipeline requires the transfer of mitochondria conferring antibiotic resistance and selection for stable hybrid cells in both uridine-deficient and antibiotic-supplemented media (Fig. 4e). Following five weeks of selection, transfer cells with permanently retained exogenous mtDNA do not require further selective growth conditions. However, and unexpectedly for the limited number of situations we have examined thus far, a recipient cell must have mismatched nDNA–mtDNA origins for exogenous mtDNA integration to occur using our MitoPunch mitochondrial transfer pipeline.

## Discussion

Prior studies of mitochondrial transfer primarily used ρ0 cells as recipients due to the relative ease of integrating exogenous mtDNA^9,31,35^. However, DNA-intercalating and DNA polymerase chain terminating drugs used to deplete mtDNA to generate ρ0 cells can have off-target nDNA effects^33,51^. Although mtDNA diseases do correlate with reduced mtDNA copy numbers in cells, no ρ0 cells exist in vivo with the exception of red blood cells^34,52–55^. ρ0 tumor cells in experimental mouse systems acquire exogenous mtDNA from host cells, which stimulates tumor progression and aggression^36,56,57^. However, the majority of mitochondrial transfer events reported in vivo usually involve some form of tissue injury and ρ+ recipient cells^58,59^. The molecular mechanisms underpinning mitochondrial transfer and permanent exogenous mtDNA integration in ρ+ cells are currently unknown and require new systems to control and study this phenomenon^3,5,8,9,13,31,60,61^.

Here, our mitochondrial transfer method improved respiration in metabolically impaired cells containing endogenous, mutant mtDNA. Other studies performed mitochondrial transplantation into ρ+ cells in vitro^29,62^ and in vivo^63–68^ for therapeutic purposes, but only observed short term changes that do not indicate permanent retention of exogenous mtDNA. For example, one study restored ATP production in an A3243G MELAS cell line using Pep-1 mediated mitochondrial transfer^62^. Mitochondrial function did improve in these dysfunctional cells after delivery, but the study was limited to four days in duration and the approach was unable to replace the endogenous detrimental mtDNA population. Another longer-term, four week study co-incubating mitochondria with WT cardiomyocytes reported only a temporary increase in mitochondrial function which, after two days, returned to the pre-transfer level of reduced respiration^28^. We observed that two weeks after transferring WT mitochondria into MELAS cells, whose respiration is similar to that of a ρ0 cell (Supplementary Fig. S1)^42^, the recipient cells survive uridine-deficient media selection without stably integrating exogenous WT mtDNA. This occurred for two independent MELAS cell lines with either matched or mismatched mitochondrial donors. In our studies, mutant mtDNA recipient cells persisted from the lack of selective pressure to integrate exogenous mtDNA. Our findings support a prior cell-to-cell mitochondrial transfer study and indicate there is a fundamental inability to transfer mitochondria into mutant ρ+ recipient cells^69^. In addition, the transfer of equal amounts of HEK293T and MELAS mitochondria into a 143BTK− ρ0 recipient cell yields co-retention of both functional and dysfunctional mtDNA. Our data suggest for currently unknown reasons that the co-introduction of multiple mitochondrial sources into a ρ0 cell that did not co-adapt to either mtDNA population results in the retention of both populations. It may be that nDNA preferentially maintains co-adapted mtDNA due to metabolic requirements^3^.

To circumvent a retention roadblock and apply an additional selective pressure to retain exogenous mtDNA, we used antibiotic-resistant mitochondria from CAP-R 501-1 cells. Previous studies have used cell fusion, co-incubation, and microinjection to deliver CAP-R mitochondria into WT cells^25,43,70^. We also achieved antibiotic-resistant mitochondrial transfer into mutant ρ+ cells. However, permanent exogenous mtDNA retention only occurred for cells with mismatched, not co-adapted mtDNA-nDNA pairs. Exogenous mtDNA retention occurred for both a mismatched ρ+ recipient and also a ρ0 recipient. For Δmt-ND4 + CAP-R 501-1 cells, there was no detectable endogenous mtDNA five weeks after mitochondrial transfer. These unexpected results suggest that the endogenous mtDNA–nDNA co-evolution somehow influences mtDNA sequence retention^71,72^. How this correlates with the retention and function of non-native mtDNA sequences in humans is unclear. Mitochondrial replacement therapies and three-person in vitro fertilization technologies used to prevent the transmission of mtDNA disorders from affected mothers have resulted in live births, suggesting the successful retention of exogenous mtDNA^73,74^. However, pathogenic mtDNA sequences have been observed in ESCs derived from these embryos, which may indicate problems with permanent exogenous mtDNA retention^75^. Understanding the biological pathways necessary to retain transferred mtDNA in vitro may therefore provide insight into improving mitochondrial replacement therapies.

Our protocol, unlike cybridization which typically requires immortal or cancerous cell fusion, can utilize replication, or ‘Hayflick’,-limited cells, to permit reprogramming to induced pluripotent stem cells. Such ‘primary’ cell recipients have a limited number of cell divisions and cannot replicate long after mtDNA depletion to generate a ρ0 cell. Our future ability in MRT in cells capable of fate transitions is of great clinical significance. Further work is needed to uncover changes to the metabolome, transcriptome, and proteome with mitochondrial transfer, to determine the extent of functional restoration in recipient cells. Our ρ+, CAP-R mitochondrial transfer pipeline could be a tool for screening recipient cells for potential factors involved in exogenous mtDNA integration.

## Methods

### Cell culture

The A3243G MELAS cybrid cell line was obtained from Carlos Moraes (University of Miami)^76^. 143BTK− ρ0 human osteosarcoma cells, cybrid cell lines with the A3243G mutation or wildtype mtDNA from the same patient, and CAP-R mouse fibroblasts (501-1) were obtained from Douglas Wallace (University of Pennsylvania)^35,45,46,77^. L929 ρ0 and mouse cybrids with a mutation in the mitochondrial-encoded NADH dehydrogenase 4^48^ (Δmt-ND4, delA10227) or mitochondrial-encoded NADH dehydrogenase 6^49,50^ (Δmt-ND6, iC13887) were obtained from Jose Antonio Enriquez Dominguez (Centro Nacional de Investigaciones Cardiovasculares Carlos III (CNIC)). HEK293T cells expressing mitochondria-label dsRed protein (pMitoDsRed, Clontech Laboratories) were generated as previously published^38^. Cells were maintained in Dulbecco’s Modified Eagle’s Medium (DMEM; Gibco, Cat. #11966-025) supplemented with 10% Fetal Bovine Serum (FBS; Omega Scientific, FB-11), 0.7 mM non-essential amino acids (Gibco, Cat. #11140-050), GlutaMAX (Gibco, Cat. #35050061), penicillin and streptomycin antibiotics (ThermoFisher Scientific, Cat. #15070063), and 50 μg/mL uridine (Sigma, Cat. #U3003). Cultured cells were routinely tested for mycoplasma with the Lonza Mycoalert Mycoplasma Detection Kit. Cells were passaged every other day and incubated at 37 °C and 5% CO_2_.

### Mitochondrial transfer workflow

Mitochondria were isolated from ~ 5 × 10^6^ donor cells using the Qproteome Mitochondria Isolation Kit (Qiagen, Cat. #37612). After isolation, around 15 µg of mitochondrial protein were suspended in 120 μL of 1 × phosphate-buffered saline (1 × PBS), pH 7.4, with calcium and magnesium and subsequently transferred into recipient cells by MitoPunch. After mitochondrial transfer, recipient cells that stably retained the exogenous mitochondria were obtained after a two-week selection in a uridine-free media that allows only cells with functional mitochondria to survive. For mitochondrial transfer experiments involving CAP-R mitochondria, transferred cell were selected in media lacking uridine and supplemented with 100 μg/mL CAP (Fisher Scientific, Cat. #22-055-125GM).

### MitoPunch

The MitoPunch is a force-based delivery tool to transfer isolated mitochondria. Using a 5 V solenoid (Sparkfun, Cat. #ROB-11015) on a threaded plug (Thor Labs, Cat. #SM1PL) inside a threaded cage plate (Thor Labs, Cat. #CP02T), this solenoid will apply a force to a deformable PDMS (10:1 ratio of Part A base: Part B curing agent, 25 mm diameter, 0.67 mm height bottom circular layer, outer diameter, 22 mm; inner diameter, 10 mm; height, 1.30 mm upper circular layer) fluid reservoir containing approximately 120 μL of isolated mitochondrial suspension. This force propels the mitochondrial suspension into ~ 200,000 adherent cells seeded onto a porous membrane with 3 µm pores (Corning, Cat. #353181) and placed above the PDMS. The solenoid is controlled using a 5 V power supply mini board (Futurlec, Cat. #MINIPOWER) and a 12 V, 3 Amp DC power supply (MEAN WELL, Cat. #RS-35-12).

### ImageStream

After MitoPunch mitochondrial transfer, cells were collected in 1.5 mL tubes and centrifuged at 1,000 × g for 5 min. The supernatant was aspirated and cells were washed 3 × with 0.5 mL PBS. After final wash, PBS was aspirated and cells were fixed with 100 μL of 4% paraformaldehyde (Thermo Fisher Scientific, Cat. #28906) for 15 min on ice. 1 mL of PBS with 5% FBS (Omega Scientific, FB-11) was added to fixative and centrifuged at 500 × g for 10 min. Supernatant was removed, cells were resuspended in PBS with 5% FBS and imaged on Amnis ImageStream^x^ MK II.

### Oxygen consumption rate flux analysis

The Seahorse Extracellular Flux Analyzer measures cellular oxygen consumption rates (OCR) to quantify mitochondrial function. Cells were plated at ~ 15,000 cells/well density in a XF96 microplate (Seahorse Bioscience, Cat. #100882-004) 24 h prior to analysis. Prior to experiments, a drug titration experiment was performed to determine the optimal concentrations of oligomycin, FCCP, and antimycin A. Treatments of 1 μM oligomycin (ATP synthase inhibitor), 0.3 μM carbonyl cyanide-4-(trifluoromethoxy) phenylhydrazone (FCCP, uncoupling agent), and 1 μM antimycin A (ubiquinone inhibitor) were added to characterize specific ETC complexes. Estimations of ATP production rates were completed using the Agilent Seahorse XF ATP Real-Time rate assay^78^. Oligomycin-sensitive respiratory rates were converted to rates of mitochondrial ATP production (ATPmito) assuming a P/O ratio of 2.73^79,80^. Glycolytic ATP rates (ATPglyco) production was estimated using the proton prediction rate (PPR) from lactate efflux provided by the XF96 Seahorse. PPR was corrected for respiratory CO_2_ acidification and geometric assay volume using values provided by Agilent. ATPglyco was estimated using a 1:1 ratio between lactate efflux and ATP generation.

### Restriction fragment length polymorphism (RFLP) analysis

Total mtDNA was isolated from cells using the DNeasy Blood and Tissue kit (Qiagen, Cat. #69504). mtDNA surrounding the CAP site were amplified by Polymerase Chain Reaction (PCR) using the following primers—forward: GAGGGTCCAACTGTCTCTTATC and reverse: TCCTTTCGTACTGGGAGAAATC. After PCR amplification, the product was digested with the MaeII restriction enzyme that cuts 5′-ACGT-3′ specifically at mtDNA sequence location 2501. The digested product was run on a 2.5% agarose gel at 100 V for 1 h and quantified using a Gene Genius bioimaging system. Last-cycle hot RFLP was not performed.

### Microscopy

Cell morphology images were taken on an Olympus CKX31 (Cat. #CKX31SF5) inspection microscope with a 4 × objective. Brightfield, DIC, and fluorescence images were obtained with a Zeiss Axio Observer Z1 microscope and Hamamatsu EM CCD camera (Cat. #C9100-02).

### Sequencing of MELAS A3243G site

To detect presence of mtDNA containing the A3243G substitution, total DNA was isolated using the DNeasy Blood and Tissue kit (Qiagen, Cat. #69504). PCR amplification of MELAS region was performed using PCR primers: forward- CCTCGGAGCAGAACCCAACCT and reverse- CGAAGGGTTGTAGTAGCCCGT. PCR products were purified using the QIAquick PCR purification kit (Qiagen, Cat #28104) and were Sanger sequenced using the forward primer.

### mtDNA qPCR quantification

Total DNA was extracted (Qiagen, Cat. # 69504) from cultured cells and mtDNA quantified using SYBR Select Master Mix for CFX (Life Technologies, Cat. # 4472942). mtDNA-encoded *MT-ND1* was amplified with the following primers: forward: CCCTAAAACCCGCCACATCT; reverse: CGATGGTGAGAGCTAAGGTC. mtDNA levels were normalized to nucleus-encoded *36B4* gene (*RPLP0*) using the following primers: forward: TGGCAGCATCTACAACCCTGAAGT; reverse: TGGGTAGCCAATCTGAAGACAGACA. qPCR was run on a BioRad CFX Thermal Cycler using the following protocol: (1) 50 °C for 2 min, (2) 95 °C for 2 min, and (3) 40 cycles at 95 °C for 10 s and 60 °C for 45 s. Samples were compared by calculating *ΔΔCT* and fold differences.

## Supplementary information

Supplementary Information.
