# Supplementary material for: Stable retention of chloramphenicol-resistant mtDNA to rescue metabolically impaired cells
Source: Sci Rep. 2020 Aug 31;10:14328. doi: 10.1038/s41598-020-71199-0 (PMC7459123; doi:10.1038/s41598-020-71199-0)
Supplement: Supplementary file 1 — Supplementary Information. [file 41598_2020_71199_MOESM1_ESM.pdf]

Supplementary Information

Stable retention of chloramphenicol-resistant mtDNA to rescue metabolically impaired cells

Emma R. Dawson[1], Alexander N. Patananan[1], Alexander J. Sercel[2], Michael A. Teitell[1-6,*]

[1]Department of Pathology and Laboratory Medicine, University of California, Los Angeles, California 90095, United States

[2]Molecular Biology Institute Interdepartmental Program, University of California, Los Angeles, Los Angeles, CA 90095, USA

[3]Eli and Edythe Broad Center of Regenerative Medicine and Stem Cell Research University of California, Los Angeles, Los Angeles, CA 90095, USA

[4]California NanoSystems Institute, University of California, Los Angeles, Los Angeles, CA 90095, USA

[5]Department of Pediatrics, David Geffen School of Medicine, University of California, Los Angeles, Los Angeles, CA 90095, USA

[6]Jonsson Comprehensive Cancer Center, David Geffen School of Medicine, University of California, Los Angeles, Los Angeles, CA 90095, USA

[*]Correspondence: mteitell@mednet.ucla.edu

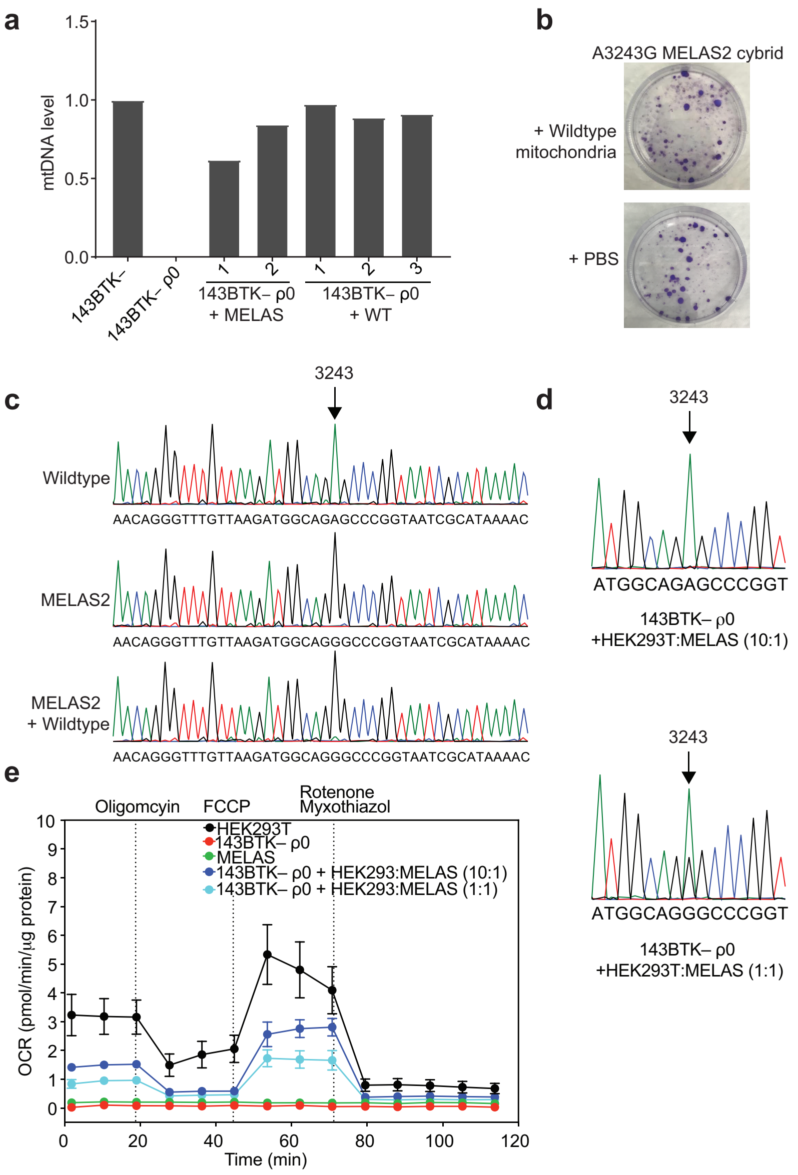

Figure S1. Selective pressure to retain mutant A3243G MELAS mtDNA.

(**a**) Quantification of mtDNA levels by qPCR in 143BTK– ρ0, 143BTK– ρ0 + MELAS clones 1 and 2, 143BTK– ρ0 + WT clones 1, 2, and 3, and the 143BTK– parent cell lines**.** The bar height denotes the average of three technical replicates. (**b**) MELAS2 + Wildtype and MELAS2 + PBS buffer cells were fixed with paraformaldehyde and stained with crystal violet to identify cell colonies. (**c**) Sanger sequencing of Wildtype, MELAS2, and MELAS2 + Wildtype. The arrow denotes mtDNA position 3243. (**d**) Sanger

sequencing of 143BTK– ρ0 + HEK293T:MELAS (10:1) and 143BTK– ρ0 + HEK293T:MELAS (1:1). Arrows denote mtDNA position 3243. (**e**) Seahorse Extracellular Flux analysis to quantify oxygen consumption rate of 143BTK– ρ0 + HEK293T:MELAS (10:1) and 143BTK– ρ0 + HEK293T:MELAS (1:1) cells. Oligomycin, FCCP, and rotenone/myxothiazol are an ATP synthase inhibitor, uncoupler, and complex I/III inhibitor, respectively. Each data point represents the average of three technical replicates and the error bar denotes standard deviation.

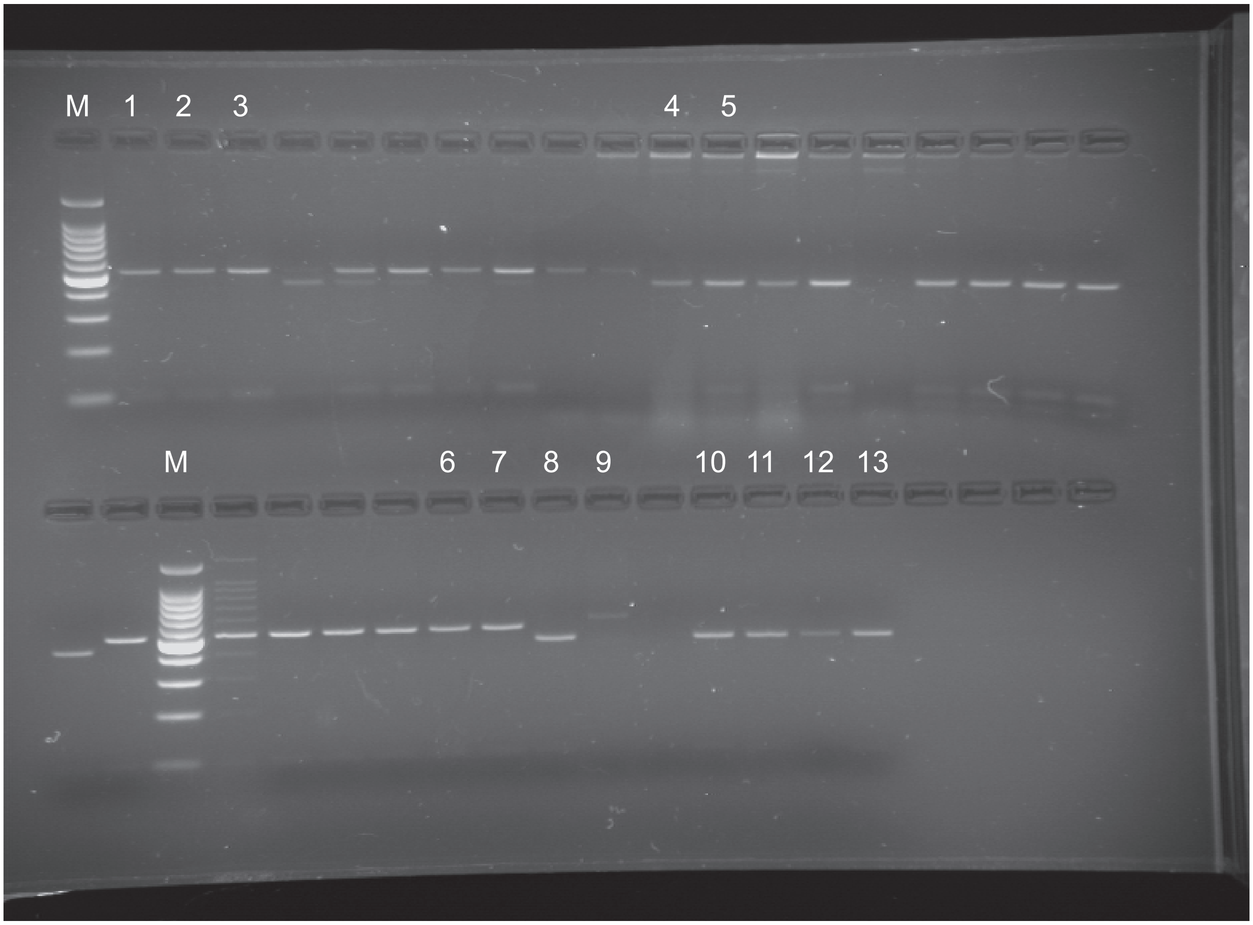

Figure S2. Uncropped gel for Figure 3e-f. PCR products post-MaeII digestion on a 2.5% agarose gel electrophoresis. Numbers on lanes correspond to the following samples: M) Marker, 1) ∆mt-ND6 + CAP-R 501-1 bulk culture 2, 2) ∆mt-ND6 + CAP-R 501-1 bulk culture 3, 3) ∆mt-ND4 + PBS bulk culture, 4) ∆mt-ND4 + CAP-R 501-1 bulk culture cultured for 5 weeks post-transfer in uridine-deficient, CAP-supplemented media, 5) ∆mt-ND4 + CAP-R 501-1 bulk culture cultured for 4 weeks in uridine-deficient, CAP-supplemented media and one additional week in CAP-deficient media, 6) ∆mt-ND6, 7) ∆mt-ND4, 8) CAP-R 501-1, 9) L929 ρ0, 10) L929 ρ0 + CAP-R 501-1, 11) ∆mt-ND4 + CAP-R 501-1 bulk culture 1, 12) ∆mt-ND4 + CAP-R 501-1 bulk culture 2, 13)∆mt-ND4 + CAP-R 501-1 bulk culture 3. Unmarked lanes on the gel are the same

transfer cell lines (L929 ρ0 + CAP-R 501-1, Δmt-ND4 + CAP-R 501-1, and Δmt-ND6 + CAP-R 501-1) cultured in different media conditions (uridine-supplemented, complete, and galactose media) at different time points throughout the selection process to optimize our selection protocol.

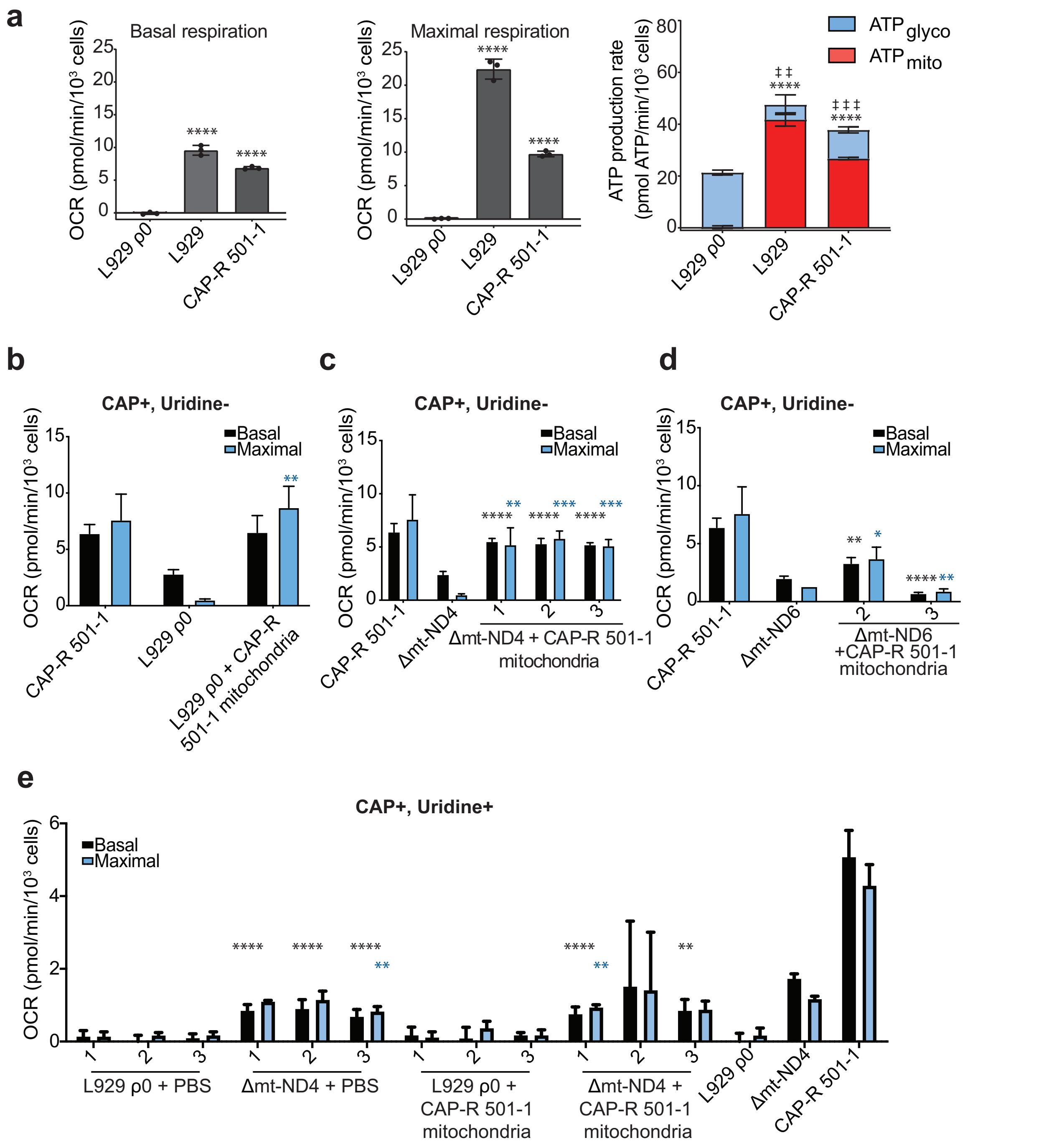

Figure S3. Basal and Maximal oxygen consumption rates corresponding to ATP production rates. (**a**) Seahorse Extracellular Flux analysis to quantify basal and maximal respiration and ATP production in L929 ρ0, L929 parent, and CAP-R 501-1 cell lines. Two-tailed, unpaired Student's t-test comparing samples to L929 ρ0. * represents

significance for ATPmito and ‡ represents significance for ATPglyco. * < 0.05, ** < 0.01, ***<0.001, ****<0.0001. ‡ <0.05, ‡ ‡ <0.01, ‡ ‡ ‡ <0.001, ‡ ‡ ‡ ‡ <0.0001. The bar height denotes the average of four replicates and the error bars are the standard deviation. (**b**) Seahorse Extracellular Flux analysis to quantify basal and maximal respiration in CAP-R 501-1, L929 ρ0, and L929 ρ0 + CAP-R 501-1. (**c**) Seahorse Extracellular Flux analysis to quantify basal and maximal respiration in CAP-R 501-1, Δmt-ND4, and Δmt-ND4 + CAP-R 501-1 clones 1, 2, and 3. (**d**) Seahorse Extracellular Flux analysis to quantify basal and maximal respiration in CAP-R 501-1, Δmt-ND6, and Δmt-ND6 + CAP-R 501-1 clones 2 and 3. For (b-d), cells had been previously cultured in uridine-deficient, CAP-supplemented media. Two-tailed, unpaired Student's t-test comparing samples to L929 ρ0, Δmt-ND4, or Δmt-ND6. The bar height denotes average of four replicates and the error bars are the standard deviation. (**e**) Seahorse Extracellular Flux analysis to quantify basal and maximal respiration in CAP-R 501-1, L929 ρ0, Δmt-ND4, L929 ρ0 + CAP-R 501-1, and Δmt-ND4 + CAP-R 501-1. Cells had been previously cultured in uridine-supplemented, CAP-supplemented media. Two-tailed, unpaired Student's t-test comparing samples to Δmt-ND4. The bar height denotes average of five replicates and the error bars are the standard deviation. For (b-e), * represents significance with * < 0.05, ** < 0.01, ***<0.001, ****<0.0001. There were no statistically significant differences when comparing the samples to L929 ρ0. Black * represents significance for basal respiration and blue * represents significance for maximal respiration.
